# Measurement characteristics of WHODAS 2.0 and WHODAS-Child: a systematic review of global psychometric studies in specific populations since 2010

**DOI:** 10.3389/fpsyt.2026.1737452

**Published:** 2026-01-26

**Authors:** Stefano Federici, Alessandro Tosti, Elena A. Russo, Lorenzo Conigli

**Affiliations:** 1 Department of Philosophy, Social & Human Sciences and Education, University of Perugia, Perugia, Italy; 2 LoveLife Associazione di Promozione Sociale (APS), Association for the Social Promotion of the Sexual Health of People with Disabilities, Perugia, Italy

**Keywords:** disability measurement, functioning, ICF, patient-reported outcome measure, PRISMA-COSMIN guideline, psychometric properties, systematic review, WHODAS 2.0

## Abstract

**Introduction:**

This global systematic review evaluated the psychometric properties, namely the reliability and validity, of all WHODAS 2.0 versions and forms, including WHODAS-Child, which has been used in specific populations since 2010, in assessing alignment with the WHO manual.

**Materials and methods:**

Following PRISMA-COSMIN guideline, electronic databases and a curated personal library were searched up to April 30, 2025. Included were 143 empirical studies on WHODAS 2.0 and WHODAS-Child in samples with specific populations, spanning 43 countries.

**Results:**

Across five data extraction forms, the mean and median values of the aggregated data regarding the measurement characteristics of WHODAS 2.0 and WHODAS-Child are reported. The 36-item version demonstrated excellent internal consistency (mean α = 0.92), while the 12-item one showed good reliability (mean α = 0.88), and the test-retest reliability was strong for both (ICC = 0.89–0.91). Factor analyses supported the six-domain structure for the 36-item version and WHODAS-Child, though more variability was noted for the 12-item version. Cultural and age-related challenges emerged, indicating the need for contextual adaptations.

**Discussion and conclusions:**

The results showed good behavior of WHODAS 2.0, albeit slightly lower than the psychometric profiles described in the Manual. WHODAS-Child, though promising, requires further validation and refinement, particularly for cross-cultural applications.

## 1 Introduction

The World Health Organization Disability Assessment Schedule (WHODAS 2.0) is a generic assessment instrument developed by the World Health Organization (WHO) to provide a standardized method for measuring health and disability (1). Its development responded to the growing need for a theoretically grounded, internationally comparable measure of disability that could be applied consistently across clinical, epidemiological, and research settings.

Being a generic measure, the instrument does not target a specific disease—unlike the Supports Intensity Scale (2) or the Autism Diagnostic Observation Schedule [ADOS (3)]; rather, WHODAS 2.0 has been designed to offer a standardized cross-cultural approach for assessing health and disability levels within the general population, regardless of any medical diagnosis (1, 4). This generic nature represents a key strength of the instrument, enabling meaningful comparisons of disability across heterogeneous health conditions and populations, like the Global Assessment of Functioning [GAF (5)] or the Vineland Adaptive Behavior Scales (6).

The independence of the evaluations provided by WHODAS 2.0 from medical diagnoses is conceptually in line with the framework of the *International Classification of Functioning, Disability and Health* [ICF (7)], which is endorsed by 191 countries as the normative system for classifying the health status of individuals (8). The ICF provides the most widely accepted and comprehensive model of disability, defined as an umbrella term denoting the negative aspects of human functioning due to the interaction between an individual’s health conditions and contextual factors manifesting as activity limitations and as restrictions in participation or problems that collide in typical life situations (at work or school, with peers, with the family, or the entire community) (7). Thus, this emphasizes the bidirectional and complex relationship between the health conditions of a person and their surrounding environmental factors, describing disability and health through a “biopsychosocial approach,” unlike previous classification systems (9).

Since disability is “complex, dynamic, multidimensional, and contested”, as stated on the *World Report on Disability* (10, p. 10), it is not possible to measure all its determinants with the same instrument at the same time. Therefore, the optimum instrument for measuring disability is one that best defines the property of disability to be measured (9, 11). In short, an elixir for measuring disability is neither possible nor desirable. That said, the unique feature of WHODAS 2.0 that distinguishes it from other disability measurements is its direct correlation to, and operationalization of, the ICF (1).

WHODAS was disseminated in 1999 in a beta version (WHODAS II) based on the WHO Psychiatric Disability Assessment Schedule (12), which was designed to assess the extent of disability associated with a psychiatric condition. A guide to the use of WHODAS II was published in 2004 (13), but the publication of *Measuring Health and Disability: Manual for WHO Disability Assessment Schedule (WHODAS 2.0)* in 2010 (1) (hereafter referred to as the Manual) marked the final version, which is referred to by the acronym WHODAS 2.0. The items of WHODAS 2.0 were selected from a large pool of ICF items that were subjected to field trials in 19 countries (1) and have been translated into more than 47 languages and dialects (9). Although other generic instruments for assessing health status can also be associated with the ICF, they do not make clear distinctions between symptom measurement, disability, and subjective appraisal. WHODAS 2.0 stands out in that it covers the ICF domains in their entirety and its applicability extends to all diseases, including physical, mental, and substance use disorders (1).

Thus, the structure and features of WHODAS 2.0 are also conceptually compatible with the atheoretical and polythetic model of the *Diagnostic and Statistical Manual of Mental Disorders, fifth edition* (DSM-5), developed by the American Psychiatric Association (14). In 1994, the DSM-IV used the GAF scale and its developed versions to assess psychiatric disability; however, when the theoretical and disability model promoted by the ICF was adopted in the DSM-5, the GAF proved inadequate for evaluating disability not as a direct consequence of an impairment. It is, therefore, not surprising that WHODAS 2.0 comes bundled with the DSM-5, with a view to replacing the more limited GAF as a standard method for assessing global disability levels for mental disorders.

This global visibility and reputation have spurred many investigations to apply it to various populations around the world, acquiring greater significance in collecting prevalence data across a variety of disabilities, disorders, and diagnoses.

WHODAS 2.0 is structured based on the activity and participation domains outlined by the ICF, offering a framework for assessing functioning across six domains: Cognition, Mobility, Self-care, Getting along, Life activities, and Participation.

Based on this structure, three versions of WHODAS 2.0 have been implemented: the 36-item version, the 12-item version, and the combined 12 + 24-item version. The 36-item and the 12-item paper-based versions were released in three forms: interview-administered, self-administered, and proxy-administered. The 12 + 24-item paper-based version was released only as an interview-administered option. The 12-item version accounts for 81% of the variance of the full 36-item version and provides results in approximately five minutes if self-administered and twenty minutes if administered via interview (1). The most widely administered versions of WHODAS 2.0 are the original 36-item and the shortened 12-item versions, both of which have been found to be reliable, valid, and sensitive to change.

According to the Manual, the 36-item version “takes into account the paid-work status of the respondent, with the 32-item being used if the respondent is not in gainful employment” (1, p. 41). The score of the 32-item WHODAS 2.0 is comparable to that of the full 36-item version.

WHODAS 2.0 can be scored using two approaches: the simple score, which sums item scores directly, and the complex score, based on item response theory, which adjusts for item difficulty. Two unresolved issues concern scoring. The WHO provides two calculation algorithms, an SPSS version in the Manual (1, pp. 58–61) and an online Excel version (15), but they yield inconsistent procedures. Moreover, the manual does not clarify how to handle missing data, particularly whether responses marked as “Not applicable” should be treated like missing data (16).

The development of the WHODAS 2.0 Manual was based on epidemiological data on disability worldwide, which revealed that WHODAS 2.0 is reliable and valid for general adult populations. However, the extent to which these psychometric properties generalize to specific clinical populations remains an open empirical question.

The WHODAS-Child was adapted for children and youth from the adult WHODAS 2.0 by the DSM-5 Impairment/Disability workgroup (17) in response to the need for a standardized instrument based on the ICF for children and youth [ICF-CY (18)]. Two versions of the WHODAS-Child were proposed (19): the 36-item patient version and the seven-item clinician version. During the WHODAS-Child 36-item adaptation process, the workgroup aimed to measure the constructs in a developmentally appropriate manner while considering the child’s environment (20). Consequently, some WHODAS 2.0 items were modified. However, at the time of the Manual’s publication, empirical evidence regarding the psychometric properties of a version of WHODAS 2.0 for children and youth was lacking because the version was still under development (Manual, p. 33).

With regard to the use of the WHODAS 2.0 Manual, two systematic reviews (9, 21) have examined the performance of this instrument, each of which had different objectives. Federici et al. (9) examined a total of 810 papers in investigating the use of the 12- and 36-item versions of WHODAS 2.0 in scientific literature from 1999 to 2015 and identified 27 research fields in which WHODAS 2.0 was used, including mental disorders, geriatrics, epidemiology, and neurology. These fields used WHODAS 2.0 in nearly 100 countries and almost 50 languages and dialects, which made it the “leading measure of disability worldwide” (9, p. 2354) at the time of writing, confirming its unidimensional structure across different research fields. However, they also recognized that the included studies had limitations, such as when self-reporting was not possible or difficult. In contrast, Saltychev et al. (21) focused on the available evidence on the psychometric properties of the self-administered 12-item WHODAS 2.0. The researchers evaluated 14 observational studies conducted on general adult populations and individuals with nonacute physical disabilities, and the study (21) revealed that the 12-item WHODAS 2.0 is multidimensional rather than unidimensional. This finding aligns with the desired characteristics of a tool of this nature, as well as with the factor structure of WHODAS 2.0, as described in the Manual. Additionally, the study found that WHODAS 2.0 may underreport actual disability experiences. Due to the absence of a ceiling effect, however, it is inadequate for use as a screening tool. Despite these limitations, Saltychev et al. (21) concluded that, based on the literature reviewed, WHODAS 2.0 has internal consistency and is reliable for test-retest situations.

As previously discussed, the adoption of WHODAS 2.0 in the DSM-5 has led to a notable increase in its visibility, and it is now used beyond epidemiological investigations and encompasses diverse patient populations across various cultural contexts.

The first objective of the present study is to verify whether the measurement characteristics (reliability: internal consistency, test-retest, and interrater reliability; and validity: convergent and concurrent, correlations, and factor structure) of any version (36-item, 12-item, 12 + 24-item) and form (interviewer-, self-, and proxy-administered) of WHODAS, as reported in psychometric studies on specific populations (e.g., with mental disorders, neurological disorders, specific disabilities, etc.), confirm or not those reported in the Manual for the general population.

A second objective of this review is to describe the measurement characteristics (reliability: internal consistency, test-retest, and interrater reliability; and validity: convergent and concurrent, correlations, and factor structure) of WHODAS-Child when administered to specific populations (e.g., children and youth with neurodevelopmental disorders, mental disorders, etc.). Due to the lack of psychometric data for this version when the Manual was published, a thorough analysis of the existing evidence is necessary to determine its applicability, advantages, and limitations in youth and pediatric populations.

## 2 Materials and methods

### 2.1 Study design

This study is a systematic review of the measurement characteristics of WHODAS 2.0 when administered to specific populations, and the measurement characteristics of WHODAS-Child. For transparent and consistent reporting of findings, data extraction and quality assessment (including a risk of bias evaluation) of the included studies were undertaken in accordance with the Preferred Reporting Items for Systematic Reviews and Meta-Analyses (PRISMA) and the Consensus-based Standards for the selection of health Measurement Instruments (COSMIN) guideline for reviews of outcome measurement instruments (OMIs) 2024 (22). PRISMA-COSMIN for OMIs 2024 is a stand-alone extension of PRISMA 2020 (23). A completed PRISMA-COSMIN checklist for systematic reviews of outcome measurement instruments is provided in **Supplementary File S1**.

### 2.2 Eligibility criteria

The present research encompasses: (i) empirical studies on psychometric properties of any version (including the Child version) or form of WHODAS 2.0 carried out from 2010 to 30 April 2025 on a specific population. For this population, we target a sample of individuals who share a common health condition, such as a mental health condition (e.g., depression, schizophrenia, trauma) or a developmental disability (e.g., autistic spectrum disorder); (ii) peer-reviewed journal articles, abstracts, and extended abstracts of international conference proceedings with a scientific committee; (iii) papers written in any language, provided they are indexed in English so that they have been indexed in the main databases and registers.

### 2.3 Information sources

A systematic search was conducted in the electronic databases of PubMed, EBSCOhost, and Scopus, employing a combination of keywords related to WHODAS 2.0, with the most recent search taking place on April 30, 2025. Furthermore, a personal library comprising 5,042 references, meticulously curated by the primary author, was thoroughly reviewed. This personal library under consideration encompasses all publications from the year 2000 to the present that feature the term “WHODAS” in their titles, abstracts, or body text.

### 2.4 Search strategy

In order to be more inclusive, an initial record search was conducted by entering search terms referring to the various acronyms and abbreviations of the World Health Organization – Disability Assessment Schedule 2.0. Therefore, the text string used was: [“WHODAS” OR “WHO-DAS” OR “WHODAS 2.0” OR “WHO-DAS 2.0” OR “Disability Assessment Schedule”], entered in the generic field of the databases (PubMed, Scopus, EBSCOhost—all databases) without further specification regarding the nature of the study or the population sample investigated. This search strategy relied exclusively on free-text keywords, as WHODAS-related terms are not consistently indexed across databases and are not systematically represented within controlled vocabularies. Therefore, no Medical Subject Headings (MeSH) terms or other thesaurus-based indexing terms were applied.

### 2.5 Selection process

The records were uploaded to the EndNote 2025 reference manager (Clarivate Analytics, 2025) and evaluated through a blind qualitative analysis by the authors AT and LC. The initial screening was performed using the article abstracts, followed by selection based on the main text documents. Methodological quality of the included studies was evaluated using the COSMIN risk of bias checklist (22).

An initial 95% agreement was reached concerning the records in question. Krippendorff’s alpha (24) was found to be 0.85, which is an adequate value for the achievement of the research work. The remaining 5% of the records were discussed among all authors until complete consensus was reached.

## 3 Results

### 3.1 Study selection

On April 30, 2025, the number of publications retrieved from each electronic database was as follows: PubMed: 1,415; Scopus: 1,867; and EBSCOhost (all databases): 1,026, for a total of 4,308 publications. After removing 2,289 duplicates, a selection process was conducted on the 2,019 remaining records, leaving 334 records sought for retrieval. Additionally, 116 papers from the primary author’s personal library that complied with the inclusion criteria were included, resulting in a total of 450 records. AT and LC screened the abstracts of 450 records to assess whether they might meet the predefined eligibility criteria. A total of 171 studies were excluded at this stage (137 from databases and registers, 34 from other methods). The same two authors then evaluated these 279 records and excluded 136 of them (92 from databases and registers, 44 from other methods) because, although they were psychometric studies of WHODAS 2.0 and WHODAS-Child, statistical analyses that met our objectives (internal consistency, test-retest and interrater reliability, convergent and concurrent validity, correlations and factor structure) were missing or only partially reported. Therefore, these studies were ineligible because the results for the outcome of interest were not reported [e.g., (25, 26)].

**Figure 1:**
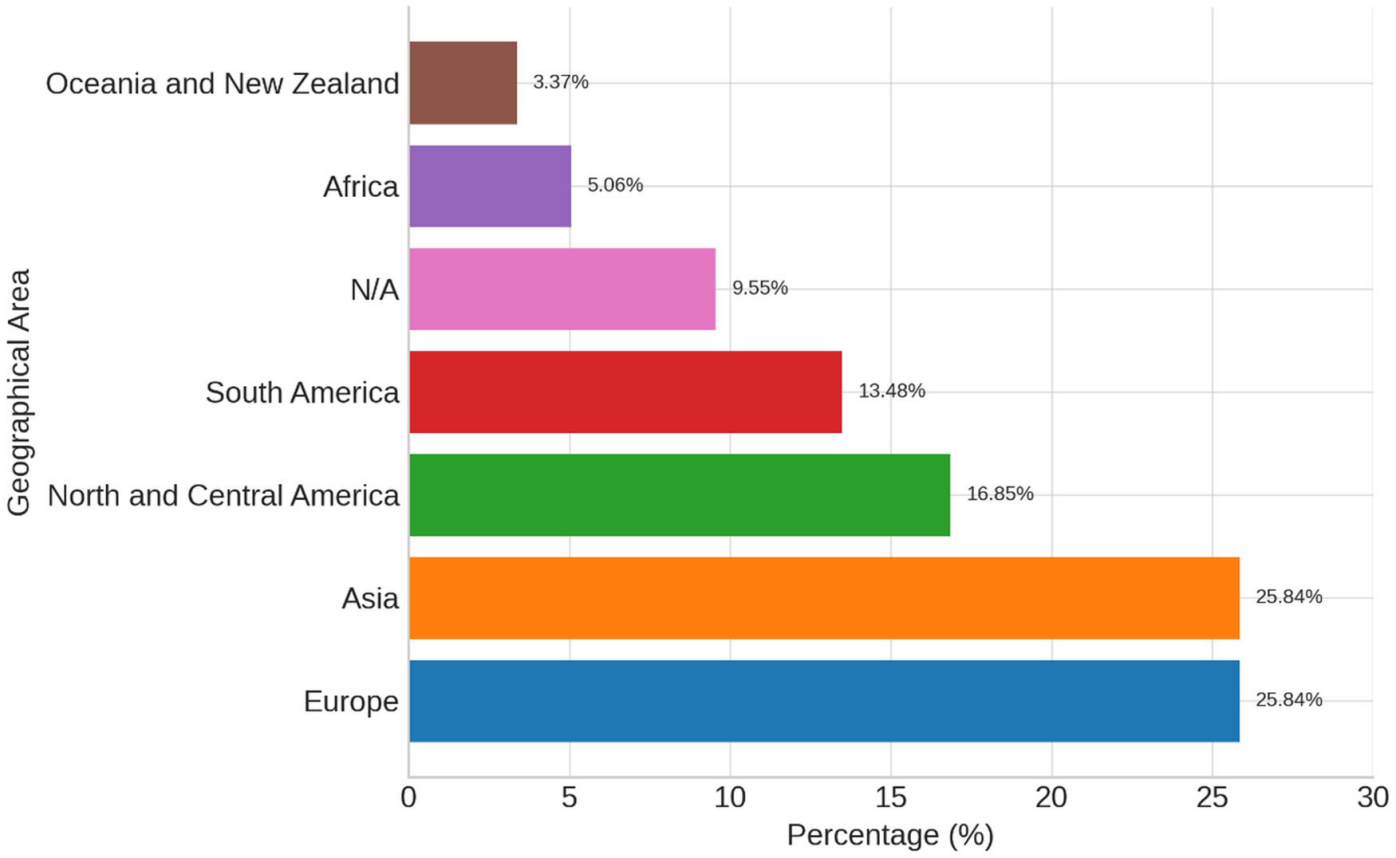
Four-phase flow diagram of the systematic review according to PRISMA-COSMIN for OMIs 2024 (22).

A total of 143 records were included in the review (20, 27–168), which underwent psychometric data extraction and qualitative text evaluation. The selection process is summarized in **Figure 1**.

### 3.2 Study characteristics

A total of 60 data items were extracted from each of the 143 included studies (e.g., research field, population, country, language, measures correlated, inter-item correlation, convergent validity, concurrent validity, factor analysis, Cronbach’s alpha, etc.) as shown in **Supplementary Table S2**. For the categories of country, language, form, and version of WHODAS 2.0, missing data are indicated with N/A in **Supplementary Table S2** if they were not specified in the included study. The “country” category refers to the location where WHODAS 2.0 or WHODAS-Child was administered to participants, regardless of their country of origin. In terms of the “study design” category, the included studies were classified into cross-sectional studies and cohort studies. We classified as cohort studies those longitudinal studies that involved the sampling of a cohort (e.g., individuals with schizophrenia, depression, anxiety, diabetes, or chronic stroke), defined as a group of individuals who share a defining characteristic, even if they do not share a common event (169). We classified those observational studies that analyzed data from a population at a single point in time as cross-sectional studies. In studies [e.g., (28, 134)] where a correlation analysis with external measures [e.g., ADOS (3)] was conducted, if the text did not specify whether the type of validity was convergent or concurrent, the correlation coefficient values (r, r², Kendall’s Tau, Kendall’s Tau-B) were reported in separate columns without further specification. With respect to the “research field” category, we have adopted the same classification used in the Federici et al. review (9). When an included study (27, 43, 76, 114, 124) investigated samples belonging to more than one research field, all fields were reported.

### 3.3 General characteristics of the studies

A total of 186 WHODAS 2.0 administrations were identified across the 143 included studies (a single included study may report results from multiple administrations). For example, Aslan Kunt et al. (31) applied all forms (interviewer-administered, self-administered, and proxy-administered) of two WHODAS 2.0 versions (36-item and 12-item). None of the included studies administered the 12 + 24-item version. Out of a total of 100 administrations (N = 186), only six (3%) utilized the WHODAS-Child version (20, 63, 66, 143, 155), making it the least widely used, while the 36-item version was employed most frequently (49%), followed by the 12-item one (42%). The self-administered form of WHODAS 2.0 was the most common for both the 12- and 36-item versions, accounting for 30% of all administrations. As for the forms used to administer WHODAS-Child, the study by Federici et al. (66) was the only one to use the seven-item clinician-administered form. The authors compared this form with data obtained from the 36-item patient-administered one and found a weak correlation (r = 0.39, p < 0.01).

**Figure 2:**
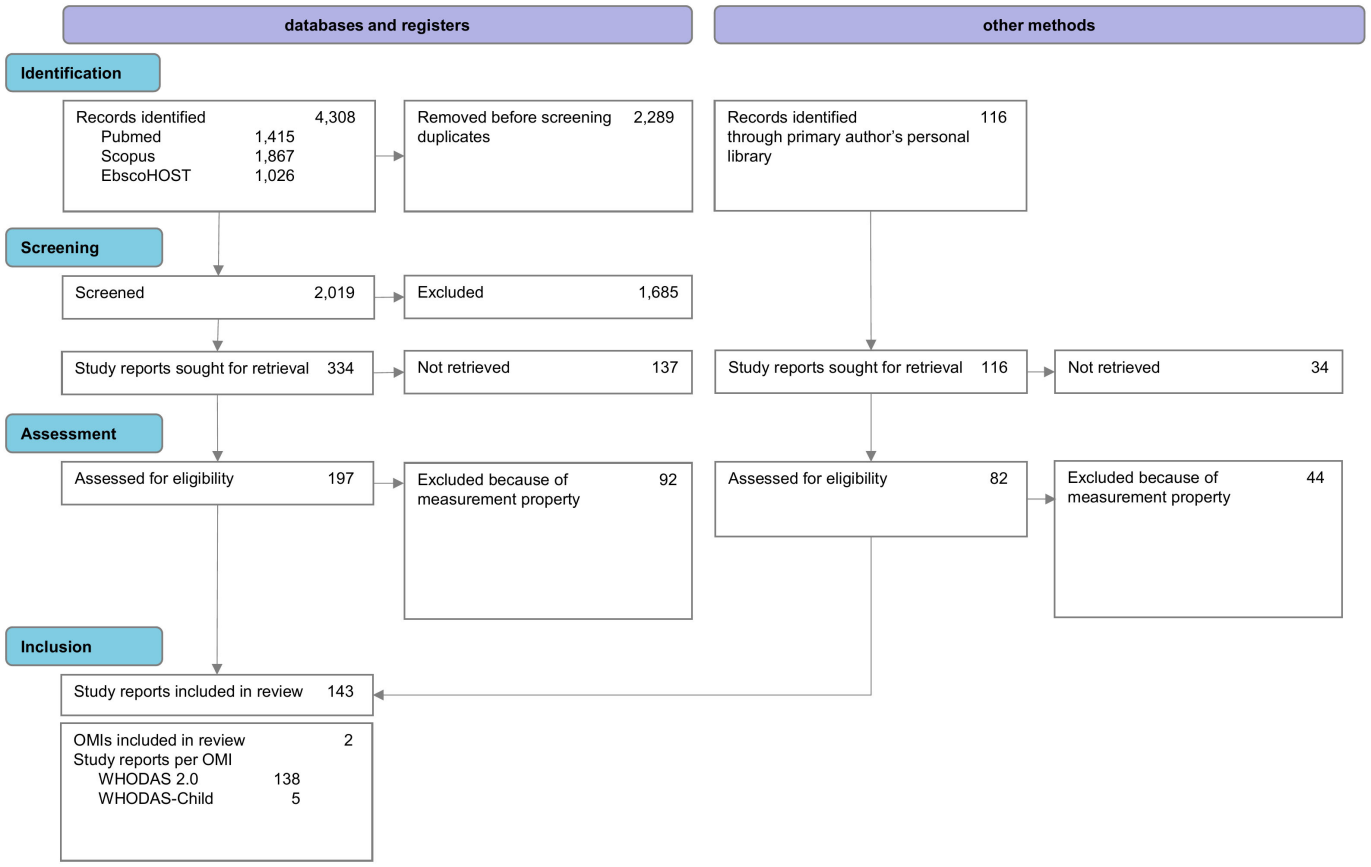
Geographical areas where the 143 included studies were conducted. Europe includes the United Kingdom and Ukraine; Asia includes the Russian Federation and Turkey.

The included studies were conducted across 43 countries on all continents (**Figure 2**), and the measure was administered in 36 languages and dialects, including all major European languages with both alphabetic and nonalphabetic systems (**Supplementary Table S2**). The countries are clustered by geographical area in **Figure 2**. Thus, Ukraine (113, 154) and the United Kingdom (163) were included among the European countries, while the Russian Federation (71) and Turkey (31) were among the Asiatic ones.

WHODAS 2.0 was applied across 16 research fields (**Supplementary Table S2**). The highest usage was observed in mental health (n = 38; 27%) (27, 28, 31, 32, 34, 38, 42, 43, 65, 72–76, 80, 81, 85, 92, 93, 97–99, 101, 108, 111, 112, 115, 116, 118, 126, 146, 149, 152, 153, 158, 159, 161, 168), disability and rehabilitation (n = 34; 24%) (36, 43, 47, 50, 51, 54, 62, 67–69, 77–79, 82, 84, 86, 87, 95, 96, 100, 102, 104, 109, 117, 121–123, 129, 133, 134, 137, 150, 157, 160), and neurology (n = 27; 19%) (30, 33, 43, 45, 48, 49, 52, 55, 57, 64, 76, 88, 107, 110, 114, 119, 120, 124, 127, 130, 138, 140, 141, 147, 162, 163, 165). These three areas together accounted for 67% (n = 96) of all included studies.

### 3.4 Measurement characteristics

Raw, disaggregated data on the measurement characteristics of WHODAS 2.0 were extracted from each of the included studies and are reported in **Supplementary Table S2**. In the following subsections, for each of the psychometric characteristics examined (reliability: Cronbach’s alpha, test-retest, interrater reliability; validity: concurrent validity, convergent validity, factor analysis), only the aggregated values of the central tendency data (mean and median) with the coefficient of variation (CV = M/SD×100) and the statistically significant results (p < 0.05) from the 143 included studies are reported (**Table 1**).

**Table 1:** Mean and median value of aggregated data regarding measurement characteristics of WHODAS 2.0 from all included studies.

WHODAS 2.0’s reliability			
	Internal consistency (Cronbach’s α)	Test-retest	Interrater reliability
12-item (mean)	Good internal consistency for all values (α = 0.88). The domain with the lowest internal consistency is Life Activities – School and Work (α = 0.68). The domains with the highest internal consistency are Mobility and Life Activities (α = 0.85).	Strong test-retest reliability (ICC = 0.91; ρ = 0.98; Cronbach’s alpha = 0.89; τb = 0.82).	Moderate interrater reliability (ICC = 0.57).
12-item (median)	Good internal consistency for all values (α = 0.89). The domain with the lowest internal consistency is Life Activities – School and Work (α = 0.68). The domain with the highest internal consistency is Mobility (α = 0.86).	Strong test-retest reliability (ICC = 0.93; ρ = 0.98; Cronbach’s alpha = 0.89; τb = 0.82).	Weak interrater reliability (ICC = 0.42).
36-item (mean)	Excellent internal consistency for all values (α = 0.92). The domain with the lowest internal consistency is Self-Care (α = 0.77). The domain with the highest internal consistency is Life Activities – Household (α = 0.88).	Strong test-retest reliability (ICC = 0.89; ρ = 0.86).	Interrater reliability ranging from moderate (r = 0.48) to strong (ICC = 0.92).
36-item (median)	Excellent internal consistency for all values (α = 0.93). The domain with the lowest internal consistency is Self-Care (α = 0.80). The domain with the highest internal consistency is Life Activities (α = 0.91).	Strong test-retest reliability (ICC = 0.89; ρ = 0.86).	Interrater reliability ranging from moderate (r = 0.5) to strong (ICC = 0.93).
WHODAS 2.0’s validity			
	Correlation	Factor analysis	
12-item (mean)	Negative moderate correlation with the SF (r = -0.58; ρ = -0.69). Negative moderate correlation with the EQ-5D (r = -0.57; ρ = -0.66). Negative moderate correlation with the TMIG Index (r = -0.5; ρ = -0.67). Positive moderate correlation with the GAF (r = 0.42).	Principal Component Analysis (PCA) 66.67% of the analysis confirms a one-factor model. Exploratory Factor Analysis (EFA) A six-factor structure or a higher-order structure has never been identified. Confirmatory Factor Analysis (CFA) 59.09% of the analysis confirms a six-factor structure or a higher-order structure with a general disability factor and six factors.	
12-item (median)	Negative correlation with the SF, ranging from moderate (r = -0.63) to strong (ρ = -0.78). Negative moderate correlation with the EQ-5D (r = -0.56; ρ = -0.66). Negative moderate correlation with the TMIG Index (r = -0.53; ρ = -0.67). Positive moderate correlation with the GAF (r = 0.6).		
36-item (mean)	Negative correlation with the SF, ranging from moderate (ρ = -0.45) to strong (r = -0.62; τ = -0.64). Negative moderate correlation with the WHOQOL-BREF (ρ = -0.58). Positive moderate correlation with the HADS (r = 0.58). Negative weak correlation with the GAF (r = -0.21).	Principal Component Analysis (PCA) 50% of the analysis confirms a one-factor model. Exploratory Factor Analysis (EFA) 21.43% of the analyses resulted in a six-factor structure. Confirmatory Factor Analysis (CFA) 73.68% of the analysis confirms a six-factor structure or a higher-order structure with a general disability factor and six factors.	
36-item (median)	Negative correlation with the SF, ranging from moderate (r = -0.56; τ = -0.64) to strong (ρ = -0.71). Negative moderate correlation with the WHOQOL-BREF (ρ = -0.59). Positive moderate correlation with the HADS (r = 0.54). Negative moderate correlation with the GAF (r = -0.4).		

SF, Medical Outcomes Study Short Form (170); EQ-5D, EuroQol – 5 dimensions (173); TMIG Index, Tokyo Metropolitan Institute of Gerontology Index (177); GAF, Global Assessment of Functioning (5); WHOQOL-BREF, World Health Organization Quality of Life-BREF (171); HADS, Hospital Anxiety and Depression Scale (178).

In addition, Summary **Table 2** reports psychometric data from WHODAS-Child. Summary **Tables 3**–**5** report psychometric data from each of the three domains most represented by the studies (mental health, **Table 3**; disability and rehabilitation, **Table 4**; and neurology, **Table 5**). Of the 143 studies included, those that did not clearly specify which version of WHODAS 2.0 (12-item or 36-item) was employed (33, 46, 57, 79, 85, 98, 114, 159, 160) were excluded from the aggregated scoring analyses. In addition, reported range values (i.e., minimum and maximum but not score points) were not included in the calculation of the mean and median aggregated data [e.g., (48, 119)].

**Table 2:** Mean and median value of aggregated data regarding measurement characteristics of WHODAS-child from all included studies (20, 63, 66, 143, 155).

WHODAS-Child’s reliability			
	Internal consistency (Cronbach’s α)	Test-retest	Interrater reliability
Mean	Good internal consistency for all values (α = 0.87). The domain with the lowest internal consistency is Participation (α = 0.67). The domain with the highest internal consistency is Life Activities – School and Work (α = 0.93).	Strong test-retest reliability (ρ = 0.83).	Strong interrater reliability (ICC = 0.88).
Median	Good internal consistency for all values (α = 0.87). The domain with the lowest internal consistency is Participation (α = 0.67). The domain with the highest internal consistency is Life Activities – School and Work (α = 0.93).	Strong test-retest reliability (ICC = 0.88; ρ = 0.83).	Strong interrater reliability (ICC = 0.88).
WHODAS-Child’s validity			
	Correlation	Factor analysis	
Mean	Positive moderate correlation with the DSM-5 levels of impairment severity/intensity support (ρ = 0.45). Positive weak correlation with the ADOS (ρ = 0.28).	Principal Component Analysis (PCA) Missing data. Exploratory Factor Analysis (EFA) 50% of the analyses resulted in a six-factor structure. Confirmatory Factor Analysis (CFA) All of the analysis confirms a six-factor structure or a higher-order structure with a general disability factor and six domains.	
Median	Positive moderate correlation with the DSM-5 levels of impairment severity/intensity support (ρ = 0.45). Positive weak correlation with the ADOS (ρ = 0.28).		

DSM-5, Diagnostic and Statistical Manual of Mental Disorders, Fifth Edition (14); ADOS, Autism Diagnostic Observational Schedule (3).

**Table 3:** Mean and median value of aggregated data measurement characteristics of WHODAS 2.0 from all included studies in the mental health field (27, 28, 31, 32, 34, 38, 42, 43, 65, 72–76, 80, 81, 92, 93, 97, 99, 101, 108, 111, 112, 115, 116, 118, 126, 146, 149, 152, 153, 158, 161, 168).

WHODAS 2.0’s reliability			
	Internal consistency (Cronbach’s α)	Test-retest	Interrater reliability
12-item (mean)	Good internal consistency for all values (α = 0.87). The domains with the lowest internal consistency are Life Activities – Household and Participation (α = 0.63). The domain with the highest internal consistency is Self-Care (α = 0.85).	Strong test-retest reliability (ICC = 0.81).	Moderate interrater reliability (ICC = 0.65).
12-item (median)	Good internal consistency for all values (α = 0.89). The domains with the lowest internal consistency are Life Activities – Household and Participation (α = 0.63). The domain with the highest internal consistency is Self-Care (α = 0.85).	Strong test-retest reliability (ICC = 0.81).	Moderate interrater reliability (ICC = 0.65).
36-item (mean)	Excellent internal consistency for all values (α = 0.93). The domain with the lowest internal consistency is Self-Care (α = 0.72). The domain with the highest internal consistency is Life Activities (α = 0.94).	Strong test-retest reliability (ICC = 0.83).	Moderate correlation when measured with Pearson’s correlation (r = 0.48).
36-item (median)	Excellent Internal consistency for all values (α = 0.93). The domain with the lowest Internal Consistency is Self-care (α = 0.73). The domain with the highest Internal Consistency is Life Activities (α = 0.93).	Strong test-retest reliability (ICC = 0.83).	Moderate correlation when measured with Pearson’s correlation (r = 0.5).
WHODAS 2.0’s validity			
	Correlation	Factor analysis	
12-item (mean)	Negative moderate correlation with the GAF when measured with Spearman’s correlation (ρ = - 0.43). Positive weak correlation with the GAF when measured with Pearson’s correlation (r = 0.42). Positive moderate correlation with the SDS (ρ = 0.69). Negative weak correlation with KIDSCREEN-27 (Kendall’s rank = -0.28).	Principal Component Analysis (PCA) All of the analysis confirms a one-factor model. Exploratory Factor Analysis (EFA) 16.67% of the analyses resulted in a six-factor structure. Confirmatory Factor Analysis (CFA) 62.50% of the analysis confirms a six-factor structure or a higher-order structure with a general disability factor and six factors.	
12-item (median)	Negative moderate correlation with the GAF when measured with Spearman’s correlation (ρ = - 0.43). Positive moderate correlation with the GAF when measured with Pearson’s correlation (r = 0.6). Positive moderate correlation with the SDS (ρ = 0.68). Negative weak correlation with KIDSCREEN-27 (Kendall’s rank = -0.28).		
36-item (mean)	Negative weak correlation with the GAF (r = -0.17).	Principal Component Analysis (PCA) 100% of the analysis confirms a one-factor model. Exploratory Factor Analysis (EFA) 50% of the analyses resulted in a six-factor structure. Confirmatory Factor Analysis (CFA) 66.67% of the analysis confirms a six-factor structure or a higher-order structure with a general disability factor and six factors.	
36-item (median)	None of the correlations are statistically significant.		

GAF, Global Assessment of Functioning (5); SDS, Sheehan Disability Scale (179); KIDSCREEN-27, KIDSCREEN-27 Quality of Life Measure for Children and Adolescents (180).

**Table 4:** Mean and median value of aggregated data measurement characteristics of WHODAS 2.0 from all included studies in the disability and rehabilitation field (36, 43, 47, 50, 51, 54, 62, 67–69, 77, 78, 82, 84, 86, 87, 95, 96, 100, 102, 104, 109, 117, 121–123, 129, 133, 134, 137, 150, 157).

WHODAS 2.0’s reliability			
	Internal consistency (Cronbach’s α)	Test-retest	Interrater reliability
12-item (mean)	Good internal consistency for all values (α = 0.88). The domain with the lowest internal consistency is Getting Along (α = 0.7). The domain with the highest internal consistency is Mobility (α = 0.85).	Strong test-retest reliability (ICC = 0.94; ρ = 0.98; α = 0.89; τb = 0.82).	Moderate interrater reliability (ICC = 0.42).
12-item (median)	Good internal consistency for all values (α = 0.89). The domain with the lowest internal consistency is Getting Along (α = 0.76). The domain with the highest internal consistency is Mobility (α = 0.85).	Strong test-retest reliability (ICC = 0.94; ρ = 0.98; α = 0.89 τb = 0.82).	Moderate interrater reliability (ICC = 0.42).
36-item (mean)	Excellent internal consistency for all values (α = 0.92). The domain with the lowest internal consistency is Life Activities – School and Work (α = 0.72). The domain with the highest internal consistency is Life Activities (α = 0.92).	Strong test-retest reliability (ICC = 0.88; ρ = 0.75).	Strong interrater reliability (ICC = 0.95).
36-item (median)	Excellent internal consistency for all values (α = 0.95). The domains with the lowest internal consistency are Self-Care and Getting Along (α = 0.8). The domain with the highest internal consistency is Life Activities – School and Work (α = 0.93).	Strong test-retest reliability (ICC = 0.89; ρ = 0.75).	Strong interrater reliability (ICC = 0.95).
WHODAS 2.0’s validity			
	Correlation	Factor analysis	
12-item (mean)	Negative moderate correlation with the SF (r = -0.65; ρ = -0.54). Positive moderate correlation with the NRS (r = 0.42; ρ = 0.41). Positive strong correlation with the ODI (r = 0.86; ρ = 0.71).	Principal Component Analysis (PCA) Missing data. Exploratory Factor Analysis (EFA) A six-factor structure has never been identified. Confirmatory Factor Analysis (CFA) 66.67% of the analysis confirms a six-factor structure or a higher-order structure with a general disability factor and six factors.	
12-item (median)	Negative moderate correlation with the SF ranging from moderate (r = -0.65; ρ = -0.54). Positive moderate correlation with the NRS (r = 0.42; ρ = 0.41). Positive strong correlation with the ODI (r = .86; ρ = 0.71).		
36-item (mean)	Moderate negative correlation with the SF (r = -0.63). Negative moderate correlation with the WHOQOL-BREF (ρ = -0.55). Negative weak correlation with the GAF (r = -0.35).	Principal Component Analysis (PCA) All of the analysis confirms a one-factor model. Exploratory Factor Analysis (EFA) A six-factor structure has never been identified. Confirmatory Factor Analysis (CFA) All of the analysis confirms a six-factor structure or a higher-order structure with a general disability factor and six factors.	
36-item (median)	Moderate negative correlation with the SF (r = -0.63). Negative moderate correlation with the WHOQOL-BREF (ρ = -0.55). Negative weak correlation with the GAF (r = -0.35).		

SF, Medical Outcomes Study Short Form (170); NRS, Numerical Pain Rating Scale (181); ODI, Oswestry Disability Index (182); WHOQOL-BREF, World Health Organization Quality of Life-BREF; GAF, Global Assessment of Functioning (5).

**Table 5:** Mean and median value of aggregated data measurement characteristics of WHODAS 2.0 from all included studies in the neurology field (30, 43, 45, 49, 52, 55, 64, 76, 88, 107, 110, 119, 120, 124, 127, 130, 138, 140, 141, 147, 162, 163, 165).

WHODAS 2.0’s reliability			
	Internal consistency (Cronbach’s α)	Test-retest	Interrater reliability
12-item (mean)	Good internal consistency for all values (α = 0.88). The domain with the lowest internal consistency is Participation (α = 0.85). The domains with the highest internal consistency are Mobility and Self-Care (α = 0.93).	Strong test-retest reliability (ICC = 0.99).	Missing data.
12-item (median)	Good internal consistency for all values (α = 0.89). The domain with the lowest internal consistency is Participation (α = 0.85). The domains with the highest internal consistency are Mobility and Self-Care (α = 0.93).	Strong test-retest reliability (ICC = 0.99).	Missing data.
36-item (mean)	Excellent internal consistency for all values (α = 0.93). The domain with the lowest internal consistency is Life Activities (α = 0.76). The domains with the highest internal consistency are Life Activities – Household and Life Activities – School and Work (α = 0.94).	Strong test-retest reliability (ICC = 0.92).	Strong interrater reliability (ICC = 0.91).
36-item (median)	Excellent internal consistency for all values (α = 0.94). The domain with the lowest internal consistency is Life Activities (α = 0.79). The domains with the highest internal consistency are Life Activities – Household and Life Activities – School and Work (α = 0.96).	Strong test-retest reliability (ICC = 0.95).	Strong interrater reliability (ICC = 0.9).
WHODAS 2.0’s validity			
	Correlation	Factor analysis	
12-item (mean)	Negative moderate correlation with the EQ (r = -0.57). Negative moderate correlation with the HSI (r = -0.59). Positive strong correlation with the WHO’s Minimal Generic Set of Domains of Functioning and Health (ρ = 0.83). Negative moderate correlation with the FIM (ρ = -0.67).	Principal Component Analysis (PCA) A one-factor model has never been identified. Exploratory Factor Analysis (EFA) A six-factor structure has never been identified. Confirmatory Factor Analysis (CFA) 50% of the analysis confirms a six-factor structure or a higher-order structure with a general disability factor and six factors.	
12-item (median)	Negative moderate correlation with the EQ-5D (r = -0.57). Negative moderate correlation with the HSI (r = -0.59). Positive strong correlation with the WHO’s Minimal Generic Set of Domains of Functioning and Health (ρ = 0.83). Negative moderate correlation with the FIM (ρ = -0.67).		
36-item (mean)	None of the correlations are statistically significant.	Principal Component Analysis (PCA) 50% of the analysis confirms a one-factor model. Exploratory Factor Analysis (EFA) 50% of the analysis confirms a six-factor structure. Confirmatory Factor Analysis (CFA) 66.67% of the analysis confirms a six-factor structure.	
36-item (median)	None of the correlations are statistically significant.		

EQ-5D, EuroQol – 5 dimensions (173); HIS, RAND-12 Health Status Inventory (183); FIM, Functional Independence Measure (184).

### 3.5 Reliability properties (internal consistency, test-retest, interrater reliability)

Among the 143 studies that were included in the analysis, 19 (13%) assessed the test-retest reliability of the 36-item version (37, 39, 42, 44, 53, 54, 56, 84, 101, 102, 104, 107, 120, 127, 130, 134, 137, 145, 157), while nine (6%) focused on the 12-item version (27, 32, 42, 55, 96, 109, 117, 129, 164). The results indicated that both the 36-item and 12-item versions demonstrated moderate to strong reliability, with the intraclass correlation coefficient (ICC) values of 0.89 and 0.93 for the median and 0.89 (CV = 8.96%) and 0.91 (CV = 9.93%) for the mean, respectively, and corresponding ρ-values of 0.86 and 0.98 for the median and 0.86 (CV = 18.09%) and 0.98 (only a data score was reported) for the mean, respectively.

A total of 104 (73%) included studies assessed internal consistency using Cronbach’s alpha, focusing on the 36-item (n = 59; 40%) (29, 31, 34, 35, 37, 39–42, 44, 47–49, 51–54, 56, 60, 61, 64, 65, 73–75, 82, 84, 90, 92, 94, 100–102, 104, 106–108, 110, 113, 115, 120, 127, 130, 133–137, 142, 145, 146, 149, 151, 157, 163–167) and 12-item (n = 41; 29%) (27, 28, 31, 32, 42, 43, 45, 50, 55, 59, 62, 68, 70, 77, 80, 81, 83, 86, 88, 91, 93, 96, 97, 99, 103, 109, 110, 117, 118, 124, 129, 131, 136, 140, 141, 144, 147, 148, 150, 155, 158, 163) versions, and WHODAS-Child (n = 5; 3%) (20, 63, 66, 143, 155). Only a small fraction used McDonald’s omega coefficient (n = 3; 2%) (34, 63, 100). Cronbach’s alpha ranged from acceptable (α ≥ 0.7) to excellent (α ≥ 0.9), with a value of 0.93 for the median and 0.92 for the mean (CV = 5.2%) for the 36-item version. For the 12-item version, the range extended from questionable (α < 0.7) to excellent, with a value of 0.89 for the median and 0.88 for the mean (CV = 7.09%). In the 36-item version, the domain with the lowest internal consistency was “Self-Care,” with a median value of 0.8 and a mean value of 0.77 (CV = 13.96%), while “Life Activities” showed the highest median value (α = 0.91). The domains “Life Activities” and “Life Activities – Household” showed the highest mean value of 0.88 (CV = 13.17%; CV = 9.82%). For the 12-item version, domain alphas generally fell between acceptable (α ≥ 0.7) and good (α ≥ 0.8), except for the “Life Activities – School and Work” domain, which was reported in only one study (80) with a questionable value (α = 0.68). WHODAS-Child showed a lower score point than WHODAS 2.0, with a score of 0.87 both for the median and mean (CV = 4.44%). Furthermore, the domains “Mobility,” “Self-Care,” and “Participation” had questionable alpha values ranging from 0.67 to 0.69. Domain-specific alpha values are detailed in **Supplementary Table S2**.

Interrater reliability was assessed in a total of nine of the included studies (20, 27, 67, 75, 107, 111, 119, 134, 165). The 36-item version yielded moderate to strong results with Pearson and ICC values, respectively, of 0.5 and 0.93 for the median and of 0.48 (CV = 0.25%) and 0.92 (CV = 0.06%) for the mean (75, 107, 111, 119, 134, 165). The 12-item version showed weak and moderate agreements when measured employing ICC index, with a median of 0.42 and a mean of 0.57 (CV = 0.49%) (27, 67). Interrater reliability for WHODAS-Child was investigated by Scorza et al., (20) who reported an ICC score of 0.88.

### 3.6 Validity properties

Of the 143 included studies, a total of 62 (43%) explored the factor structure of WHODAS 2.0 (36-item and 12-item versions) using the following analyses: Six studies (4%) (52, 76, 78, 118, 156, 162) ran a principal component analysis (PCA), 31 studies (22%) (28, 32, 39, 42, 50, 52, 53, 59, 65, 71, 76, 78, 84, 109, 113, 115, 118, 120, 122, 123, 128, 129, 135, 138–140, 144, 148, 162, 165, 167) an exploratory factor analysis (EFA), 39 studies (27%) (27, 28, 34, 42, 43, 45, 51, 53, 59, 62, 65, 67–69, 73, 74, 80–82, 89, 90, 93, 94, 101, 106, 110, 120, 123, 128, 130, 135, 136, 138, 150, 153, 158, 161, 162, 164) a confirmatory factor analysis (CFA), and three studies (2%) (124, 144, 155) ran a confirmatory tetrad analysis (CTA). Only three of the included studies (52, 113, 115) that used the EFA statistical technique matched the six-domain structure of the 36-item version proposed by the Manual. Conversely, the structure of the 12-item version was not found to replicate the six-domain structure as proposed in the Manual for the 36-item version when the EFA technique was adopted. When the CFA technique was employed on the 36-item version (n = 19), a factorial structure as proposed in the Manual was found 14 (74%) times in the included studies (42, 51, 65, 73, 74, 82, 90, 94, 101, 106, 120, 128, 130, 161). The factor structure of the 12-item version replicated the six-domain structure as proposed in the Manual for the 36-item version. This was demonstrated in 13 out of 22 included studies that employed it (59%) (43, 45, 62, 67–69, 80, 81, 110, 136, 153, 158, 164).

Three included studies investigated the factor structure of WHODAS-Child: Díaz-Castro et al. (63) used EFA and CFA; Scorza et al. (20) employed CFA; and Umucu et al. (155) utilized EFA and CTA. As regards the WHODAS-Child factor structure, Díaz-Castro et al. (63) confirmed the six-domain structure, whereas Umucu et al. (155) reported a two-factor structure.

As shown in **Supplementary Table S2**, WHODAS 2.0 was correlated with health measures, indices, functional health, well-being, and quality of life. The values below (Pearson’s correlation [r], Spearman’s correlation [ρ], Kendall’s Tau [τ], and Kendall’s Tau-b [τb]) refer to the four measures most commonly correlated with the 36- and 12-item versions of WHODAS 2.0, at a significance level of less than 0.05: the Medical Outcomes Study Short Form [SF (170)], the World Health Organization Quality of Life scale [WHOQOL (171)], the Global Assessment of Functioning scale [GAF (172)], and the European Quality of life – 5 dimensions instrument [EQ-5D (173)]. The 36-item version demonstrated negative correlations with several outcome measures, considering both median and mean values. Specifically, in relation to the SF (37, 40, 44, 54, 61, 102, 113, 145, 154), the median correlations were r = -0.56, τ = -0.64, and ρ = -0.71, while the mean correlations were r = -0.62, τ = -0.64, and ρ = -0.45, with CV ranging from 27.59% to 127.6%. With regard to the WHOQOL-BREF (171), the median correlation was ρ = -0.59 and the mean was ρ = -0.58, with a CV of 28%. Finally, for the GAF (5), the median correlation was r = -0.4 and the mean was r = -0.21, with a CV of 216.84%.

A positive Pearson correlation was identified between the 12-item version and the GAF (42, 72, 97), as indicated by a median value of 0.6 and a mean value of 0.42 (CV = 107.45%). However, a negative median (r = -0.63; ρ = -0.78) and mean (r = -0.58; ρ = -0.69; CV ranging from 16.91% to 21.5%) correlation was shown with the SF (83, 91, 96, 109). Likewise, EQ-5D showed a negative correlation with WHODAS 2.0, as indicated by the median (r = -0.56; ρ = -0.66) and mean values (r = -0.57, CV = 9.99%; ρ = -0.66) (45, 58, 70, 131) (**Table 1**).

Federici et al. (66) also found positive Spearman correlations between the seven-item WHODAS-Child form for clinicians and the 36-item WHODAS-Child form for patients and the ADOS, with respective correlations of ρ = 0.27 and ρ = 0.29 (**Table 2**).

### 3.7 Summary tables

The following two tables report the mean and median values of the aggregated data regarding the measurement characteristics of WHODAS 2.0 (**Table 1**) and WHODAS-Child (**Table 2**) from all the included studies. The subsequent three tables report the mean and median values of the aggregated data for the three most represented research fields: mental health (**Table 3**), disability and rehabilitation (**Table 4**), and neurology (**Table 5**). The following cutoff criteria were applied to all data reported in the five tables: Cronbach’s alpha: 0.5 > x = unacceptable; 0.6 > x ≥ 0.5 = poor; 0.7 > x ≥ 0.6 = questionable; 0.8 > x ≥ 0.7 = acceptable; 0.9 > x ≥ 0.8 = good; x ≥ 0.9 = excellent (174); ICC: x ≤ 0.4 = weak; 0.75 > x > 0.4 = moderate; x ≥ 0.75 = strong (175); Pearson’s correlation (r), Spearman’s correlation (ρ), Kendall’s Tau (τ), and Kendall’s Tau-b (τb): 0.1 > x = trivial; 0.39 ≥ x ≥ 0.1 = weak; 0.69 ≥ x ≥ 0.4 = moderate; 0.9 ≥ x ≥ 0.7 = strong (176).

## 4 Discussion

The present systematic review examined 143 (20, 27–168) studies that investigated the measurement properties of WHODAS 2.0 (36-item, 12-item, 12 + 24-item) and its adaptation for children and youth (WHODAS-Child) as reported in psychometric studies on specific populations from 2010 up to today. The aim was to verify whether or not these characteristics confirm those reported in the Manual for the WHO Disability Assessment Schedule (WHODAS 2.0) by Üstün et al. (1) for the general population.

### 4.1 Test-retest reliability

The mean and median values for test-retest reliability of the 12-item version indicate excellent performance of the measure. These data are not reported in the Manual, which refers only to the 36-item version. Compared to the 36-item version, the test-retest reliability values do not confirm those in the Manual, which reports stronger values (ICC = 0.98) than the mean and median values (ICC = 0.89) obtained from our aggregate data, which show good performance. These findings indicated that the test demonstrated adequate performance. The frequency distribution of the aggregated scores shows low variability (CV < 10%), and studies that deviate negatively from the mean have ICC values that never fall below the acceptable range for both the 12-item (27) and the 36-item (127, 137) scales.

Of the five included studies of WHODAS-Child, only one (20) measured test-retest validity twice, with both cases showing a strong performance. This finding is not comparable to that of the Manual, which at the time of publication was still investigating the use of the children and youth version.

### 4.2 Cronbach’s alpha

The Manual reports Cronbach’s alpha scores for the 36-item WHODAS 2.0, both for each of the six domains (cognition, mobility, self-care, getting along, life activities, and participation) and for total scores. No such scores are reported for the 12-item version. Only about 3% of the studies included reported all seven alpha values [e.g., (44, 54, 62, 75, 163, 165)]. With regard to the internal consistency of the total score, the mean and median show higher values for the 36-item version than for the 12-item one. The mean and median values of the 36-item version replicate the Manual values with very low variability (CV < 10%). Some studies report only acceptable values [e.g., (35, 135)], although none of these interpret them as compromising the internal reliability of the measurement.

The behavior of the measure for each of the six domains differs from the total score. In fact, the aggregate scores from the reviewed studies for each of the six domains are lower than the alpha scores reported as excellent in the Manual. The mean scores range from acceptable (self-care and getting along) to good (cognition, mobility, life activities, and participation), and the median scores are all in the good range. However, there is some variability (23.1% ≥ CV ≥ 7.51%) across studies in the Cronbach’s alpha values of the six domains. For example, the results obtained by Badu et al. (34) on a sample of consumers of mental health services in Ghana essentially replicate the internal consistency values of the Manual, with the authors claiming that WHODAS 2.0 is a reliable measure with satisfactory psychometric properties. In contrast to the previous study, de Castro (60) found that the internal consistency for the getting along domain had an unacceptable Cronbach’s alpha.

The internal consistency scores for the 12-item version, which are good for the total score in terms of mean and median and for the six domains range between questionable and good, cannot be compared with those in the Manual because they are missing. Ćwirlej-Sozańska et al. (55), who conducted a study on a sample of people with Huntington’s disease, reported Cronbach’s alpha values for the entire scale of 0.97 and for individual domains ranging from 0.95 to 0.97. In contrast, Holmberg et al. (81) found questionable scores for half of the domains in patients with psychotic disorders, although the overall alpha was considered good.

As with the previous 12-item version, comparable data with the Manual for WHODAS-Child are not available. The five studies included here (20, 63, 66, 143, 155) agree on a good value for the total score. The values of the individual domains have only been calculated by Federici et al. (66) and Díaz-Castro et al. (63), both of which converge on values that range from acceptable for cognition to excellent for life activities, while diverging for the remaining domains.

### 4.3 Factor structure

According to the Manual, factor structure analyses conducted using confirmatory factor analysis on the 36-item version of the scale “revealed a two-level hierarchical structure, with a general disability factor feeding into the six domains” (1, p. 20). In addition, the same Manual states that each of the six domains has a unidimensional structure. This structure thus distributed (1 + 6) was found in the vast majority of the cases (74%) in the reviewed studies (34, 42, 51, 53, 65, 73, 74, 82, 90, 94, 101, 106, 110, 120, 128, 130, 135, 161) that have ascertained the factor structure. However, five studies (34, 53, 110, 135, 161) did not find this distribution. Williams et al. (161) advocate a seven-factor solution by suggesting that the life activities domain be split into two subscales because it contains items of a different nature: household items and work/school items. The study by Park et al. (110) is of the same opinion. Less consensus on a 6 + 1 structure was found using EFA (39, 52, 53, 65, 76, 78, 84, 113, 115, 120, 128, 135, 165, 167).

The factorial structure of the 12-item version remains debated, as already noted by Saltychev et al. (21) in their systematic review of the psychometric properties of the 12-item version self-administered in the general population and in people with nonacute physical causes of disability. The authors stated that the conclusions of the review by Federici et al. (9) on the factorial structure of the 12-item version were too optimistic, a structural weakness that is particularly evident when the EFA technique is used. In this case, none of the 18 times for which this analysis was performed replicated a six-factor structure (28, 32, 42, 50, 59, 71, 109, 118, 122, 123, 129, 138–140, 144, 148, 162).

A six-factor structure was found in the WHODAS-Child factor analyses when examined with CFA (20, 63) and EFA (63, 155).

### 4.4 Concurrent validity

The mean and median values of correlations with health measures (e.g., SF, WHOQOL) obtained from the included studies refer only to the total scores obtained on WHODAS 2.0. In contrast, in the Manual, correlation scores are calculated only for each domain. Nevertheless, according to our results, there is generally a negative satisfactory correlation with the main related health measures. This supports the specificity of the WHODAS 2.0 disability construct with respect to related but not coincident health domains, confirming the findings as reported in the Manual. The same can be said for the correlations between WHODAS-Child and the ADOS (3) carried out by Federici et al. (66).

Findings on concurrent validity therefore indicate that WHODAS 2.0, when compared with widely used generic health and quality-of-life measures such as the SF-36 or WHOQOL, is designed to primarily capture activity limitations and participation restrictions rather than perceived health status or subjective well-being. This conceptual focus, grounded in the ICF-based biopsychosocial model of disability, makes WHODAS 2.0 particularly suitable for contexts in which disability is operationalized beyond symptom severity or health-related quality of life. At the same time, it provides a theoretical explanation for the moderate correlations observed with generic health and quality-of-life measures.

### 4.5 General considerations

Since the publication of the Manual, WHODAS 2.0 has seen increasing use in various research fields, most frequently in mental health, followed by disability and rehabilitation, and neurology. These findings reinforce the role of WHODAS 2.0 as a core measure of disability in the DSM-5 and the notion that it effectively operationalizes the ICF model, especially in fields where a biopsychosocial perspective is crucial (1).

Notably, the 12-item version is frequently used, likely because of its practicality. Despite its conciseness, its psychometric properties are consistent with those of the 36-item version, though its reliability indices are slightly lower. These findings support using the 12-item version in large-scale screenings and surveys where limitations are critical while maintaining satisfactory validity and reliability levels (32).

Both the 12-item and 36-item versions were primarily self-administered, except in research on disability and rehabilitation, where the interview form prevailed. This suggests that the method of administration is often adapted to the characteristics of the sample.

Several studies [e.g., (63, 74, 84, 106, 167)] have criticized the clarity, adaptability, and feasibility of adapting the wording to low-income or non-Western contexts, which significantly compromises the interpretability and comprehensibility of certain items, particularly those assessing household, work, school, and sexual activities. These findings underscore the necessity of culturally adapting WHODAS 2.0 and imply that, in certain contexts, some items may necessitate revision or contextual explanation to preserve construct validity. For instance, the feasibility of item 4.5, “sexual activity,” was low, probably due to living conditions and the private nature of sex in Asian (53), Italian (185), and conservative cultures (82, 165), or to psychological factors in the case of breast cancer patients (167).

Similarly to cultural adaptation, feasibility issues arise with regard to specific populations, such as respondents with intellectual disabilities and schizophrenia. Chiu et al. (53) claim that people with intellectual disabilities have difficulty recognizing the meaning of dignity in their lives (item D6.3 of the 36-item version: “How much of a problem did you have living with dignity because of the attitudes and actions of others?”). With regard to people with schizophrenia, although the instrument exhibits suitable psychometric properties in terms of reliability and validity (111, 149), respondents tend to misjudge items, reporting general disability values that are underestimated compared to the general population (81).

In the case of WHODAS-Child, Federici et al. (66) recommend increasing the number of studies to allow for a larger population of children with disabilities, which would make it possible to develop normative data for clinical use. Convergent and discriminant validity studies would better define the WHODAS-Child construct as a generic measure of disability based on the ICF-CY version (18) model and allow it to be correlated with other specific measures of child functioning and health. Finally, Federici et al. (66) recommend reconsidering the reintroduction of an item on sexual activity in WHODAS-Child. This item was present in the original adult version but was replaced by “getting along with his/her teachers” (item D4.5 of the 36-item patient form) in the same position and domain. A measure designed to assess the functioning of young people that does not also consider sexual behavior (e.g., masturbation) is not credible. For example, individuals with developmental disabilities may engage in socially inappropriate masturbation, which can put them or others at risk of harm (186). Moreover, these behaviors have such a strong impact on the social and family environment that they may restrict youth participation in everyday situations.

It should be noted that the 12- and 36-item versions for adults were used in studies with adolescent and young participants [e.g., (67–69, 99, 153, 155, 162)]. The results of the psychometric analysis confirm that both versions perform well with this age group. Furthermore, when the scores on the functioning of children with mental disabilities (obtained via the self-administered form) were compared with the values of their functioning as assessed by their parents (obtained by administering the proxy form), the children reported greater functional impairment than their parents did (99). The reasons for this discrepancy are unclear, though it’s possible that parents are more susceptible than their children to acquiescence bias and an idealistic distortion of their children’s functioning. The same discrepancy was found in Federici et al. (66), who compared the scores of treating physicians and parents of children with autism spectrum disorder. The low correlations between parents’ and professionals’ opinions confirm the differences in their health perspectives. This reinforces the importance of considering both perspectives to gain a richer, more complex view of a child’s health and functioning.

As inherently subjective measurements, WHODAS 2.0 and WHODAS-Child cannot distinguish between actual individual functioning and perceived disability. This makes it unclear whether the observed activity limitations and participation restrictions reflect objective individual capacity or personal perception of one’s own functioning. As with any health measure, this reaffirms the need to combine subjective measures of individual functioning with those of health experts, such as treating physicians, which ensures that the internal view of disability when assessing healthcare or evaluating medical strategies is not misleading (187).

### 4.6 Limitations

This review has several limitations. First, the inclusion criterion requiring the selection of empirical studies on the psychometric properties of any version or form of WHODAS 2.0 from 2010 may have led to the exclusion of studies that used the old acronym, WHODAS II, to refer to the current WHODAS 2.0. However, only studies for which it was impossible to verify the version used were excluded. For example, Luciano et al. (188) was excluded because it was impossible to determine whether the version used (WHODAS II) coincided with the 2010 version (WHODAS 2.0). Park et al. (110), on the other hand, was included because, despite the inappropriate use of the acronym WHODAS II, it was clear that they were referring to the current version, WHODAS 2.0. The same applies to the 2010 date limit. Studies produced before 2010 that used the WHODAS 2.0 version, as published in the official release of the Manual in 2010, may have been excluded.

Second, due to differences in the study designs, sample sizes, populations investigated, and analysis methods of the included studies, a meta-analysis of the data was not possible, although an aggregation of the data from the included studies was conducted.

Third, the available correlation data were limited to associations between the total scores of WHODAS 2.0 and the total or subscale scores of external measures. Many included studies also reported correlations with individual WHODAS 2.0 domains; however, these were not considered in this study.

Finally, the summaries of values reported by each included study did not consider data that reported score ranges rather than point scores, and eliminating these data may have affected the calculation of the aggregate medians and means reported in **Tables 1** and **3**–**5**.

## 5 Conclusion

This systematic review provides a comprehensive synthesis of the psychometric properties of WHODAS 2.0 across diverse populations and research fields. The findings confirm the overall reliability and validity of both the 36- and 12-item versions. The results showed good behavior on the part of the WHODAS 2.0 36-item version, albeit slightly lower than the psychometric profiles described in the Manual (1). The tool showed particularly strong measurement characteristics in populations with mental health conditions, neurological disorders, and rehabilitation needs, which together accounted for over two thirds of the reviewed studies. In these groups, internal consistency, test-retest reliability, and construct validity were consistently high, thereby supporting the utility of WHODAS 2.0 in clinical and research contexts.

While the one general disability and six domains proposed in the Manual were largely supported, some studies suggested alternative factorial solutions, particularly for the 12-item version. The limited use and evaluation of WHODAS-Child underscore the need for further research to establish its psychometric robustness and clinical applicability. Cultural and contextual factors emerged as critical considerations, especially in non-Western and low-income settings, emphasizing the importance of making adaptations that are sensitive to culture. Overall, WHODAS 2.0 remains a valuable tool for assessing disability within the ICF framework; however, future studies should address its limitations in specific populations, such as children, individuals with intellectual disabilities, and those from culturally diverse backgrounds, and further explore its use in youth assessments, as this will enhance its global applicability and clinical utility.

Finally, as the present review showed, WHODAS 2.0 is widely used in clinical and research settings across various countries; however, its relevance extends beyond clinical and research contexts to administrative ones as well. Several countries, including Australia, Canada, Germany, and Sri Lanka, have integrated WHODAS 2.0 into their disability assessment protocols or health service guidelines, often aligning with the ICF framework. Italy was the first country to formally adopt WHODAS 2.0 through national legislation—Legislative Decree No. 62/2024 (189)—which grants it binding legal status in the evaluation of civil invalidity and disability. This marks the first time a patient self-assessment has been incorporated into the Italian national social security evaluation process, reaffirming the gradual shift towards a biopsychosocial and patient-driven approach to assessing a person’s functioning and disability.

## Data Availability

The datasets presented in this study can be found in online repositories. The names of the repository/repositories and accession number(s) can be found in the article/**Supplementary Material**.
