# Supplementary material for: Measurement characteristics of WHODAS 2.0 and WHODAS-Child: a systematic review of global psychometric studies in specific populations since 2010
Source: Front Psychiatry. 2026 Jan 26;17:1737452. doi: 10.3389/fpsyt.2026.1737452 (PMC12883983; doi:10.3389/fpsyt.2026.1737452)
Supplement: Supplementary file 1 [file DataSheet1.pdf]

PRISMA - COSMIN OUTCOME Measurement Instruments

Full reports

Section and Topic	#	Checklist item[a]	Location
**TITLE**			
Title	1	Identify the report as a systematic review and include as applicable the following (in any order): outcome domain of interest, population of interest, name/type of OMIs of interest, and measurement properties of interest.	Title page
**ABSTRACT**			
BACKGROUND			
Objectives	2.1	Provide an explicit statement of the main objective(s) or question(s) the review addresses.	accomplished
METHODS			
Risk of bias	2.2	Specify the methods used to assess risk of bias in the included studies.	accomplished
RESULTS			
Included studies	2.3	Give the total number of included OMIs and study reports.	accomplished
Synthesis of results	2.4	Present the syntheses of results of OMIs, indicating the certainty of the evidence.	accomplished
DISCUSSION			
Interpretation	3	Provide a general interpretation of the results and important implications.	accomplished
**OPEN SCIENCE**			
Support	4	Describe sources of financial or non-financial support for the review, and the role of the funders in the review.	Section funding
Competing interests	5	Declare any competing interests of review authors.	Conflict of interest statement
Availability of data, code, and other materials	6	Report which of the following are publicly available and where they can be found: template data collection forms; data extracted from included studies; data used for all analyses; analytic code; any other materials used in the review.	Supplementary Table S2 or https://doi.org/10.13140/RG.2.2.19770.66244
**INTRODUCTION**			
Rationale	7	Describe the rationale for the review in the context of existing knowledge.	accomplished
Objectives	8	Provide an explicit statement of the objective(s) or question(s) the review addresses and include as applicable the following (in any order): outcome domain of interest, population of interest, name/type of OMIs of interest, and measurement properties of interest.	accomplished
**METHODS**			
Followed guidelines	9	Specify, with references, the methodology and/or guidelines used to conduct the systematic review.	Section 2.1
Eligibility criteria	10	Specify the inclusion and exclusion criteria for the review.	Section 2.2
Information sources	11	Specify all databases, registers, preprint servers, websites, organizations, reference lists and other sources searched or consulted to identify studies. Specify the date when each source was last searched or consulted.	Section 2.3
Search strategy	12	Present the full search strategies for all databases, registers, and websites, including any filters and limits used.	Section 2.4

PRISMA - COSMIN OUTCOME Measurement Instruments

Full reports

Section and Topic	#	Checklist item[a]	Location
Selection process	13	Specify the methods used to decide whether a study met the inclusion criteria of the review, e.g., including how many reviewers screened each record and each report retrieved, whether they worked independently, and if applicable, details of automation tools/AI used in the process.	Section 2.5
Data collection process	14	Specify the methods used to colect data from reports, e.g., including how many reviewers collected data from each report, whether they worked independently, any processes for obtaining or confirming data from study investigators, and if applicable, details of automation tools/AI used in the process.	Sections 2.5 and 3.1
Data items	15	List and define which data were extracted (e.g., characteristics of study populations and OMIs, measurement properties' results, and aspects of feasibility and interpretability). Describe methods used to deal with any missing or unclear information.	Section 3.2<br>Supplementary Table S2
Study risk of bias assessment	16	Specify the methods used to assess risk of bias in the included studies, e.g., including details of the tool(s) used, how many reviewers assessed each study and whether they worked independently, and if applicable, details of automation tools/AI used in the process.	Section 2.5
Measurement properties	17	Specify the methods used to rate the results of a measurement property for each individual study and for the summarized or pooled results, e.g., including how many reviewers rated each study and whether they worked independently.	Sections 3.4 and 3.7
Synthesis methods	18a	Describe the processes used to decide which studies were eligible for each synthesis.	Section 2.5
	18b	Describe any methods used to synthesize results.	Sections 3.4 and 3.7
**RESULTS**			
Study selection	19a	Describe the results of the search and selection process, from the number of records identified in the search to the number of study reports included in the review, ideally using a flow diagram. If applicable, also report the final number of OMIs included and the number of study reports relevant to each OMI.	Section 3.1
	19b	Cite study reports that might appear to meet the inclusion criteria, but which were excluded, and explain why they were excluded.	Section 3.2
OMI characteristics	20a	Present characteristics of each included OMI, with appropriate references.	Introduction
	20b	If applicable, present interpretability aspects for each included OMI.	Introduction
	20c	If applicable, present feasibility aspects for each included OMI.	Introduction
Study characteristics	21	Cite each included study report evaluating one or more measurement properties and present its characteristics.	Sections 3.2 and 3.3
Risk of bias in studies	22	Present assessments of risk of bias for each included study.	Section 2.5
Results of individual studies	23	For all measurement properties, present for each study: (a) the reported result and (b) the rating against quality criteria, ideally using structured tables or plots.	Supplementary Table S2
Results of syntheses	24	Present results of all syntheses conducted. For each measurement property of an OMI, present: (a) the summarized or pooled result and (b) the overall rating against quality criteria.	Section 3.7, Tables 1–5
Recommendations	25	If appropriate, make recommendations for suitable OMIs for a particular use.	Sections 4.5 and 5
**DISCUSSION**			
Discussion	26a	Provide a general interpretation of the results in the context of other evidence.	Section 4
	26b	Discuss any limitations of the evidence included in the review.	Section 4.6
	26c	Discuss any limitations of the review processes used.	Section 4.6

Section and Topic	#	Checklist item[a]	Location
	26d	Discuss implications of the results for practice, policy, and future research.	Section 5

From: Elsman EBM, Mokkink LB, Terwee CB, Beaton D, Gagnier JJ, Tricco AC, et al. Guideline for reporting systematic reviews of outcome measurement instruments (OMIs): PRISMA-COSMIN for OMIs 2024. *Quality of Life Research* (2024), doi: https://doi.org/10.1007/s11136-024-03634-y.
